# Integrating Human Intestinal Organoids into FDA's New Approach Methodologies for Drug Discovery

**DOI:** 10.1002/advs.202522276

**Published:** 2026-03-02

**Authors:** Debarun Patra, Ibrahim M. Sayed, Souhrid Mukherjee, Honit Piplani, Aida Habtezion, Michael J. Rosen, Jospeh C. Wu

**Affiliations:** ^1^ Stanford Cardiovascular Institute Stanford University School of Medicine Stanford California USA; ^2^ Division of Cardiovascular Medicine Department of Medicine Stanford University School of Medicine Stanford California USA; ^3^ Greenstone Biosciences Palo Alto California USA; ^4^ Cibus Therapeutics Palo Alto California USA; ^5^ Division of Gastroenterology and Hepatology Department of Medicine Stanford University School of Medicine Stanford California USA; ^6^ Division of Pediatric Gastroenterology Hepatology and Nutrition Department of Pediatrics Stanford University School of Medicine Stanford California USA; ^7^ Department of Radiology Stanford University School of Medicine Stanford California USA

**Keywords:** drug discovery, FDA's new approach methodologies, gastrointestinal toxicity, human intestinal organoids, inflammatory bowel disease

## Abstract

Reliance on cell lines and animal models for toxicity testing has long been the cornerstone of preclinical drug discovery for intestinal diseases, but the physiology, genetics, and disease etiology of animals differ significantly from those of humans. Species‐specific differences contribute to high drug attrition rates at early stages of clinical trial, particularly in complex disorders such as inflammatory bowel disease (IBD), in which such a rate is over 85%. Regulatory shifts by the FDA with the Modernization Act 2.0 and subsequent New Approach Methodologies (NAMs) guidance are accelerating the transition toward human‐relevant systems by emphasizing AI‐integration, organoid‐based assays, and organ‐on‐chip technologies. Human intestinal organoids (HIOs) have emerged as transformative tools that faithfully replicate the architecture, function, and cellular diversity of the human gut. Advances in the HIOs allow study of drug absorption, metabolism, and toxicity in a dish, providing a bridge between in vitro assays and clinical outcomes to offer new opportunities for improving the prediction of toxicokinetics and pharmacokinetics. Emerging clinical trials employing patient‐derived intestinal organoids (PDO) underscore their potential to bridge preclinical and clinical drug development. HIOs, as a disease model, fit well with the FDA's NAMs roadmap, and they will improve drug safety assessment and reduce the use of animal models.

## Graphical abstract

Illustration summarizes how human intestinal organoids (HIOs) are becoming transformative in preclinical research. Preclinical drug discovery pipelines often rely on animal models for ADMET studies, even though interspecies ADME gaps, poor external validity, and high attrition rates are common. The FDA's New Approach Methodologies (NAMs) suggest a robust, human‐relevant platform for evaluating drug safety and efficacy. With the patient‐derived intestinal organoids and advanced microphysiological systems, drug testing will be more effective. In addition, in silico toxicity prediction shortens the first layer of screening, and the robust HIO models are handy for permeability testing, pharmacokinetic assessment, and toxicity assessment. HIOs as NAMs support the shift toward animal‐free, precision‐driven toxicology and drug discovery mandated by the FDA. Illustration created with BioRender.

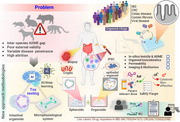

## 1 Introduction

Bringing a new drug to market heavily depends on efforts put into preclinical research. The preliminary data on a drug's toxicity, efficacy, and safety were primarily obtained through animal experiments. However, over the last few decades, regulatory and funding agencies have made continuous efforts to reduce reliance on animals for preclinical drug safety and efficacy testing [1, 2]. Preclinical animal models are often rodents, dogs, minipigs, and non‐human primates, which share both similarities and differences in physiology with humans [3]. Most drugs failed in the earliest phase of clinical trials because they appeared safe in preclinical animal testing, highlighting the massive gap in translation between animals and humans [4, 5, 6]. Suggesting that the traditional practice of drug testing in animals become less faithful in duplicating the human response. Humans are highly heterogeneous in physiology, microbiome, and genetics; thus, preclinical animal models often fall short at modeling human conditions and serve as poor predictors of human safety [7, 8, 9]. In addition, there are significant interspecies differences in absorption, distribution, metabolism, and excretion (ADME) parameters, target gene expression, and immunological profiles [10, 11, 12], which cannot be replaced by better species selection and cross‐species testing [13].

The regulatory and funding authorities recognized the limitations in recent times and initiated the utmost required shifts from animal to human‐relevant models. In 2022, groundbreaking regulatory shifts came from the FDA with its Modernization Act 2.0 [14], which encourages testing results from in vitro advanced human models and opens a much‐needed path for drug approval applications. The authorization of this act was validated in 2025 using New Approach Methodologies (NAMs) [15, 16]. With this gear shift, regulatory submission now allows results from human‐relevant models, cell‐based assays, artificial intelligence (AI), computational models, and microphysiological systems (MPS). In preclinical safety assessments, NAMs will help refine and standardize testing methods and, potentially, increase their wider acceptance across the pharma industry in the future under the FDA's vision [17, 18].

FDA's NAMs will soon reshape the drug discovery field, especially in intestinal diseases. The intestine is a central organ of human physiology for drug absorption and metabolism [19]. Orally administered drugs pass through the organ before entering the bloodstream [20]. Aside from animals, preclinical research on intestinal diseases has previously relied on immortalized human cell lines (such as Caco‐2 cells) to assess drug absorption and transport [21, 22]. However, their efficiency remained limited due to poor metabolic enzymatic activity and cellular complexity compared to the human intestine [23, 24]. To overcome these limitations, multidisciplinary efforts are employed to develop human‐based models that better reflect intestinal metabolism, transport, and toxicity with the realistic biological conditions [25].

**FIGURE 1:**
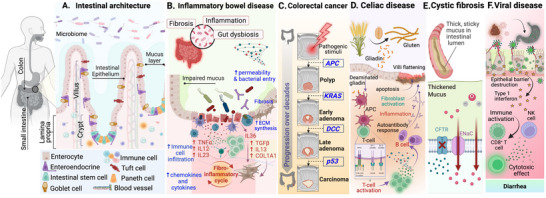
Intestinal architecture and the pathological mechanisms underlying various intestinal diseases. (A) Intestinal Architecture: the cellular composition of the intestinal epithelium, including enterocytes, goblet cells, Paneth cells, enteroendocrine cells, and immune cells. The internal structure of crypts shows the role of the microbiome and the mucus layer in maintaining intestinal barrier function. (B) Inflammatory Bowel Disease (IBD): A chronic inflammatory and fibrotic processes that characterize the disease. Highlighted the impaired mucus layer, dysbiosis, immune cell recruitment, and the fibro‐inflammatory cycle, exacerbating the disease pathogenicity. (C) Colorectal Cancer: Illustration showing the progression of colorectal cancer. Accumulating genetic mutations leading to polyp formation and the development of invasive carcinoma. (D) Celiac Disease: The autoimmune response triggered by gluten in genetically predisposed individuals is shown, leading to villous atrophy, inflammation, and T‐cell activation. (E) Cystic Fibrosis: CFTR mutations impair mucus production and ion transport, leading to thickened mucus and compromised nutrient absorption, increasing susceptibility to infections. (F) Viral Disease: The depiction of the effects of viral infections on the intestinal epithelium, including epithelial barrier destruction, immune activation, and the cytotoxic effects of viral infection, which commonly result in diarrhea. Figure created with BioRender.

The past two decades have seen remarkable progress in stem cell biology, tissue bioengineering, and microfluidics, including the development of 3D human intestinal organoids, derived from adult intestinal stem cells, induced pluripotent stem cells (iPSCs), or explants. With these advances, it is possible to generate human intestinal epithelium with crypt‐villus architecture, having multiple epithelial cell types (colonocytes, Paneth cells, tuft cells, enteroendocrine cells, goblet cells, etc.) that express relevant transporters and enzymes [26]. By directly modeling human tissue, advanced human intestinal organoids in a dish provide exceptional opportunities to test drug toxicity, study pharmacokinetics, and investigate disease biology [27]. Moreover, PDOs capture the human variability and disease phenotypes in conditions such as inflammatory bowel disease (IBD), celiac disease, colorectal cancer (CRC), viral infections, and cystic fibrosis [28, 29, 30] (Figure 1).

In particular, IBD underscores the pressing need for more human‐based models in drug discovery. Despite more than 700 active clinical trials registered, only about 15% of IBD drug candidates that enter phase I are ultimately approved [31, 32]. The cost and timeline for discovering a single drug and bringing it to market typically exceed $2.5 billion and span a decade or longer, with nearly half of that investment spent during preclinical development [33, 34, 35]. Phase III failures remain frequent, and clinical outcomes, such as mucosal healing, often plateau at around 30%–40% even for market leader biologics (e.g., adalimumab, infliximab, and ustekinumab) [32, 36, 37]. To improve these outcomes, the ability to integrate HIOs in an advanced multiple organ‐on‐chip platform allows the dynamic modeling of drug absorption, metabolism, and toxicity [38]. Already, organoid‐based assays outperform traditional cell lines in predicting drug‐induced gastrointestinal injury and chemical toxicity [39]. To advance drug testing toward more predictive, high‐throughput approaches with NAMs, combining measurements of morphological alterations, functional changes, and multi‐omics profiling will be explored [25, 40]. By integrating with a deep learning model, human‐based in vitro outputs, and AI‐powered virtual screening, NAMs will uncover safer, effective compounds at early stages of drug discovery [41].

As the FDA's regulatory shift is reshaping preclinical drug discovery and safety testing, we reviewed the emergence of HIOs as the central player in NAMs, recalibrating preclinical and early‐phase drug development for intestinal diseases. We had dived into the registered clinical trials on intestinal diseases, including PDOs for studying disease etiology and testing drugs. Moving forward, the current and upcoming challenges associated with NAMs in preclinical research on intestinal disease and highlighted advances that offer solutions.

## 2 Why are Human Intestinal Organoids Vital in Preclinical Drug Discovery?

The human GI tract runs from the oral cavity to the rectum, not only digesting and absorbing foods but also, in its lower section (the intestine), hosting trillions of microbes that shape immune responses and maintain delicate metabolic balance, immunity, and drug responses [41, 42, 43]. Interestingly, interindividual variability in the gut microbiome is well documented, and dietary alterations and environmental factors often alter the microbiota [44, 45, 46], making the intestine a challenging organ to study using traditional preclinical models. In particular, dietary composition and its complexity uniquely shape the human gut, and they are far more complex than those of other preclinical animal models [47, 48, 49]. The intestinal epithelium acts as a barrier that protects the host from luminal microbes and dietary antigens [50]. In the small intestine, the epithelium is organized into finger‐like villi, which maximize surface area for absorption, and crypts, which harbor proliferating stem and progenitor cells [51, 52]. The crypt base is enriched with highly proliferative intestinal stem cells that give rise to mature epithelial cell lineages [53]. The cell composition of the intestinal epithelium includes enterocytes, multiple secretory cells, including goblet cells, enteroendocrine cells, and tuft cells [54]. Paneth cells are also interspersed at the crypt base and secrete antimicrobial peptides and support the stem cell niche [55]. Moreover, intestinal dynamics are supported by neurons, vascular cells, immune cells, and mesenchymal cells [56, 57, 58]. (Figure 1). Suggesting that modeling the human intestine is far more complex and challenging to model with a traditional setup. Culturing PDOs is superior and enables the investigation of complex intestinal diseases and testing drug safety, efficacy, and metabolism.

## 3 Advancements in Human Intestinal Organoids

In vitro human intestinal models are highly advanced for disease modeling and drug testing [59, 60]. Starting with epithelial cell lines (such as Caco‐2, HT‐29, and T84) used for intestinal transport and drug permeability [61, 62], now replaced by Human adult intestinal stem cells or iPSCs derived *3D epithelial organoid culture systems* [63, 64]. These HIOs retain a self‐organized structure, forming crypt‐villus domains, exhibit multilineage differentiation, and physiological expression of nutrient transporters and metabolic enzymes [65, 66]. Intestinal organoids generated from patients' biopsies are called PDOs, preserving patients' pathogenic signatures, genetic, and epigenetic marks when cultured in a dish [67]. An advanced patient‐derived “organoid village” system allows population‐based drug testing and toxicity screening in a dish [68, 69, 70].

**FIGURE 2:**
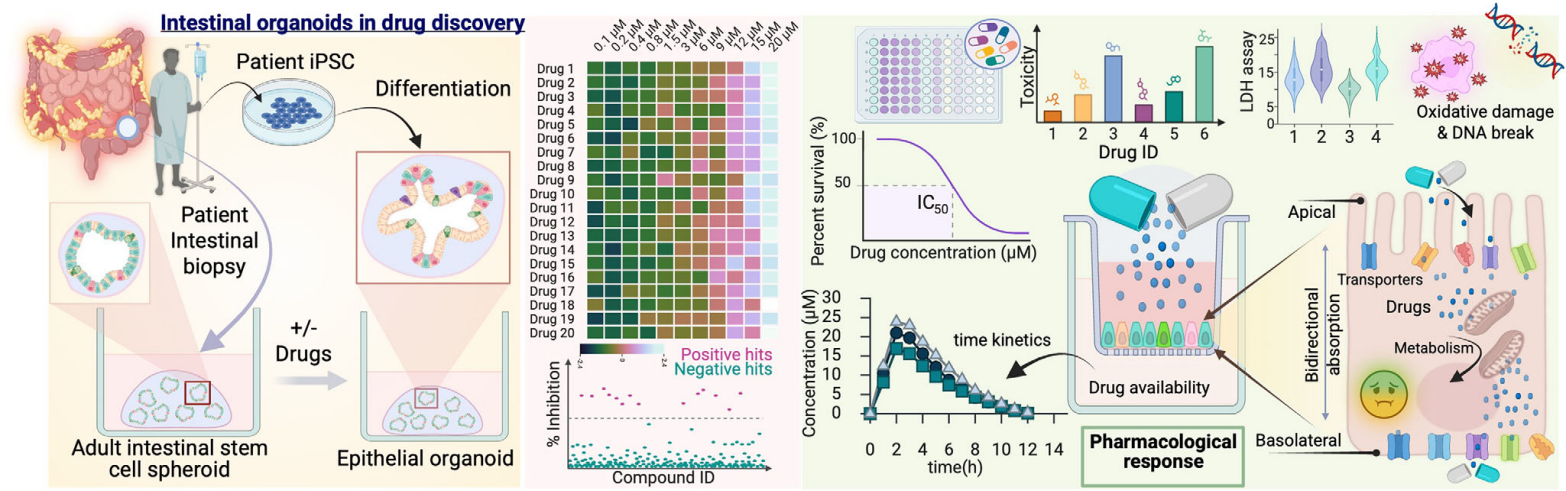
Human intestinal organoid for drug discovery and pharmacological assessment. Illustration shows the use of human intestinal organoids (HIOs) as preclinical models to evaluate drug toxicity, pharmacokinetics, and efficacy. It is now possible to bridge the gap between cell‐based assays and in vivo models, providing a physiologically relevant system for predicting early toxicity, pharmacokinetics, and therapeutic response during drug development. Organoids can be differentiated from adult intestinal stem cells or iPSCs. High‐throughput screening assays with HIOs measure cell viability and functional aspects and are often used for toxicological measurements, including oxidative stress, DNA damage, and lactate dehydrogenase (LDH) release assays. Investigation of drug absorption, metabolism, and transport across the apical‐to‐basolateral surfaces of the intestinal epithelium showed realistic bidirectional drug movement. Figure created with BioRender.

HIOs generated from intestinal stem cells (ISCs) and from iPSCs differ in several aspects but hold potential in disease modeling and drug discovery (Figure 2). The key elements are: 1) availability and sourcing, 2) disease phenotype fidelity, 3) applications in personalized medicine, and 4) robustness and scalability for drug discovery. For example, iPSC‐HIOs offer an unlimited cell source after generating an iPSC line and do not require repeated access to patient tissue. In contrast, PDOs depend on the availability and quality of primary intestinal biopsies, which can be limited in specific patient populations. PDOs become advantageous for modeling chronic intestinal diseases as they retain epigenetic features, tissue‐specific transcriptional programs, and the regional identity of the donor intestine [71, 72]. These features potentiate them to be well‐suited for predicting individual drug responses and toxicity, and they set the standard for developing personalized medicine. In contrast, iPSCs undergo epigenetic resetting during reprogramming, which might limit their ability to capture disease‐associated epigenetic states, but they are extremely valuable for studying genetic disorders (such as cystic fibrosis [73], microvillus inclusion disease [74], congenital enteropathies [75], and monogenic IBD [76]) and for systematic genetic manipulation. iPSC‐HIOs are a superior choice for greater standardization and suitability for high‐throughput screening, compared to PDOs. Suggesting the platforms are complementary, however, the selection of the appropriate system should be guided by the biological question, disease context, and translational objective (Table 1).

**TABLE 1:** Comparison of human intestinal organoids (HIOs) generated from adult intestinal stem cells and iPSCs.

Feature	Adult ISC‐Derived Organoids (PDOs)	iPSC‐Derived Organoids
Source	Patient biopsies	Induced pluripotent stem cells
Epigenetic fidelity	Preserves tissue‐specific epigenetic marks	Epigenetic resetting during reprogramming
Disease modeling	Excellent for chronic diseases (e.g., IBD, CRC)	Ideal for genetic disorders and developmental studies
Personalized medicine	High potential (patient‐specific)	Limited unless combined with genetic editing
Scalability	Moderate (depends on biopsy availability)	High (theoretically unlimited cell source)
High‐throughput use	Less suited	Well suited
Current adoption	More common in translational and clinical studies	Growing in developmental biology and rare disease research

Functional aspects of the human intestinal epithelium, such as barrier integrity (TEER, FITC‐dextran permeability), can be measured with simpler 2D monolayers that are derived from PDOs [77, 78, 79, 80]. Further integration with microfluidic organ‐on‐chip technologies reproduces key mechanical and biochemical cues, such as shear stress, peristaltic motion, and oxygen gradients [81, 82, 83]. With these advancements, it is possible to capture dynamic epithelial responses to microbial metabolites, immune mediators, and drugs. Co‐culture approaches that incorporate immune cells, fibroblasts, neurons, or commensal microbes now enable interrogation of the gut‐immune‐microbiome axis under both homeostatic and inflammatory conditions [84, 85]. Moreover, multi‐omics profiling (transcriptomic, proteomic, metabolomic, and epigenomic) of PDOs has revealed disease‐specific molecular signatures in IBD and other intestinal diseases [86, 87, 88, 89]. Ranging from 2D monolayers to 3D patient‐derived HIOs and organ‐on‐chip systems, each in vitro model contributes unique mechanistic insights and translational value (Table 2).

**TABLE 2:** In vitro human intestinal models.

Model Type	Composition	Key Functional Features	Applications	Advantages	Challenges	Refs.
2D immortalized cell lines (e.g., Caco‐2, HT‐29, T84)	Human CRC‐derived epithelial monolayers	Polarized epithelium; basic barrier & transporter function	Drug permeability, absorption, and transport assays	Simple, cost‐effective, high throughput, standardized	Limited cell diversity; lack of crypt‐villus structure, mucus layer, immune or microbial components	[208]
Primary intestinal epithelial cultures	Directly isolated epithelial sheets or crypts from human or animal intestine	Retain donor‐specific phenotype for short term; limited proliferation	Short‐term toxicity testing; nutrient uptake studies	Human tissue relevance	Short‐lived, low scalability, donor variability	[209]
3D adult stem cell derived enteroids	Lgr5+ adult intestinal stem cells embedded in Matrigel; growth in Wnt3A‐R‐spondin‐Noggin niche	Self‐organize into crypt‐villus domains; multilineage differentiation (enterocytes, goblet, Paneth, enteroendocrine, tuft cells)	Modeling IBD, infection, CF, intestinal regeneration; mucosal toxicity	Recapitulate in vivo architecture, long‐term expansion, patient‐derived	Closed lumen limits apical exposure; batch variability of ECM	[175]
iPSC‐derived human intestinal organoids	iPSC >definitive endoderm > midgut >HIO with epithelium + mesenchyme	Mesenchymal & epithelial compartments; mimic developmental maturation	Developmental biology, genetic disease modeling, drug metabolism	Unlimited source, genetic engineering possible	Immature phenotype, lack of immune/microbiota components	[210]
Organoid‐derived monolayers (2D from 3D)	Dissociated organoids cultured on transwell membranes	Apical accessibility; measure TEER, permeability, cytokine responses	Barrier integrity, xenobiotic transport, host–pathogen interactions	Amenable to high‐throughput; compatible with microelectrode arrays	Loses 3D spatial gradients, limited long‐term maintenance	[79]
Co‐culture systems (immune, microbiota, fibroblasts)	Organoids combined with immune cells or commensal microbes	Models gut‐immune gut‐microbiota crosstalk; cytokine/immune signaling	IBD, infection, immunotoxicity	Captures host‐microbe dynamics; physiologic relevance	Complex optimization; batch variability; biosafety	[105, 211]
Microfluidic organ‐on‐chip systems	Organoid or epithelial monolayers cultured under flow, shear stress, cyclic stretch	Recreates mechanical & biochemical cues of intestine	Dynamic drug absorption & toxicity; microbiome studies; PK modeling	Continuous perfusion; real‐time sampling; integration with sensors	Fabrication cost; throughput limitations; standardization pending	[212]
Multi‐organ MPS	Coupled intestinal and hepatic or neuron or immune MPS modules	Sequential first‐pass metabolism, immune activation	Systemic exposure modeling, drug–drug interaction assessment	Captures inter‐organ PK/TK interactions	Requires advanced microengineering and validation	[192, 194, 195]

## 4 Organoid‐Based Assessment of Early Toxicity, Pharmacokinetic Profile, and Drug Discovery

HIOs have emerged as powerful and essential tools for defining quantitative toxicokinetic parameters in the early stages of drug discovery, expressing tight junction proteins ZO‐1, E‐cadherin, Occludin, and Claudins [63, 79, 90]. Their physiological barrier integrity allows researchers to study selective permeability and drug absorption, and how it interacts with the intestinal wall [91, 92]. These HIOs also express a wide range of transporters that regulate drug absorption and bioavailability, including SGLT1, OATP1A2, ASBT, PepT1, P‐gp, BCRP, and MRP1 [91, 92, 93, 94]. In addition, they express genes and enzymes that regulate lipid metabolism, such as APOA1, APOA4, LPL, and MTTP, making them useful for studying how dietary fat or nutrient composition influences drug uptake and metabolism [95]. This combination of transport and metabolic activity enables HIOs to mimic the functional intestine far more closely than previous models (Figure 2).

In a functional study, intestinal organoids have shown the ability to distinguish between drugs with high, moderate, and low permeability; these include highly permeable (propranolol, digoxin, and ketoprofen), moderately permeable (atenolol), and poorly permeable (valaciclovir) drugs [91]. For example, in a drug absorption testing model, HIOs were used to test 19 commercially available drugs, producing excellent performance with drugs having high permeability coefficients [92]. Intestinal spheroid‐derived 2D monolayers are capable of bidirectional transport measurement of P‐gp substrate and BRCP substrate drugs. For instance, sulfasalazine, a commonly used drug in IBD and a BCRP substrate, showed significantly higher movement only from basolateral to apical side in the absence of P‐gp/BCRP inhibitors [96]. Other studies demonstrated HIOs function in active drug metabolism with expression of phase I and phase II metabolism enzymes, including cytochrome P450 (CYP3A4, CYP2C9, CYP2C19), Carboxylesterase 2 (CES2), and conjugation enzymes (UGT1A1, UGT1A3, SULT, and NAT‐1) [91, 95]. In these studies, HIOs were used to measure key drug metabolites that reflect their metabolic activity.

It has been shown that the levels of hydroxytestosterone and hydroxycelecoxib, the primary phase I metabolites of testosterone and celecoxib, increased steadily over time, indicating that the CYP3A4 and CYP2C9 enzymes were active within the human duodenal organoids. It confirms the HIOs support normal oxidative metabolism. Similarly, the metabolite levels of estradiol‐3‐glucuronide, 7‐Hydroxycoumarin sulfate, and N‐Acetyl‐5‐aminosalicylic acid (phase II metabolites of estradiol, 7‐Hydroxycoumarin, and Mesalazine) are increased in a time‐dependent manner, suggesting the activity of UGT1A1, SULT, and NAT‐1 [91]. In another study, iPSC‐derived intestinal organoids were utilized with an apical‐out orientation to evaluate how rifampin modulates metabolic and transport pathways relevant to pharmacokinetics. Rifampin upregulates the expression of CYP3A4 and MDR1, mediating xenobiotic metabolism and efflux. Rifampin treatment also altered the kinetic behavior and reduced the permeability of co‐administered drugs [92], suggesting that HIOs can effectively model complex drug–drug interactions.

HIOs are more efficient in predicting drug absorption and intestinal exposure, providing a stronger link between in vitro results and systemic toxicity in humans [26, 97, 98]. “Organoid villages” generated from diverse donors (e.g., derived from healthy controls and individuals with IBD, celiac disease, or colon cancer) will allow for capturing interindividual variability, and estimates population‐level risk predictions in drug discovery by utilizing advanced pharmacogenetics tools such as single‐cell multi‐omics [68, 99]. Downstream from this pipeline, demultiplexing is performed using demuxlet, which identifies parallel cultures of multiple donor organoids, maintains donor specificity, and controls batch effects through demultiplexing of pooled samples [100, 101]. In addition, functional readouts such as cell viability, permeability, and mitochondrial respiration can be measured before and after‐drug treatment [102]. These results help identify the 10%–20% effective dose (EC_10_/_20_), where early toxic effects first appear. When combined with in vitro to in vivo extrapolation (IVIVE) and physiologically based pharmacokinetic models, the data can be used to estimate equivalent doses in humans [26, 103].

HIOs are valuable platforms for testing the safety and toxicity of drugs. Researchers can measure several toxicology parameters on this model, such as cellular cytotoxicity, DNA/RNA damage, and DNA breaks, which enable the identification of potential genotoxic properties of drugs [104, 105]. HIOs have been utilized to study the host‐microbe interactions, such as the impact of *H*. *pylori*, *Fusobacterium nucleatum*, and *Escherichia coli*, on the development and progression of colorectal cancer and inflammatory bowel disease [105, 106, 107, 108]. To assess the gut toxicity of therapeutic compounds, a similar approach can be applied that compares the drug's half‐maximal inhibitory concentration (IC_50_) with its maximum serum concentration (C_max_). An IC_50_/C_max_ ratio greater than 30 generally indicates a relatively low gastrointestinal risk. In contrast, a lower ratio predicts potential gut injury or diarrhea [109, 110]. Nifedipine showed minimal intestinal toxicity, whereas colchicine caused pronounced epithelial damage [94, 111]. For example, one recent study evaluated therapeutic compounds Gefitinib, SN‐38, and OTX015 on HIOs and observed a dose‐dependent cytotoxicity and epithelial barrier disruption, whereas ribavirin resulted in no such effect [91]. These data aligned with the in vivo findings showing that Gefitinib, SN‐38, and OTX015 caused diarrhea and gastrointestinal toxicity [91]. These findings provide evidence that HIO‐based assays can serve as a valuable platform assessment of drug safety and evaluating the kinetic profile of drugs (Figure 2).

## 5 FDA's New Approach Methodologies Fast‐Tracking GI toxicity Investigations

**FIGURE 3:**
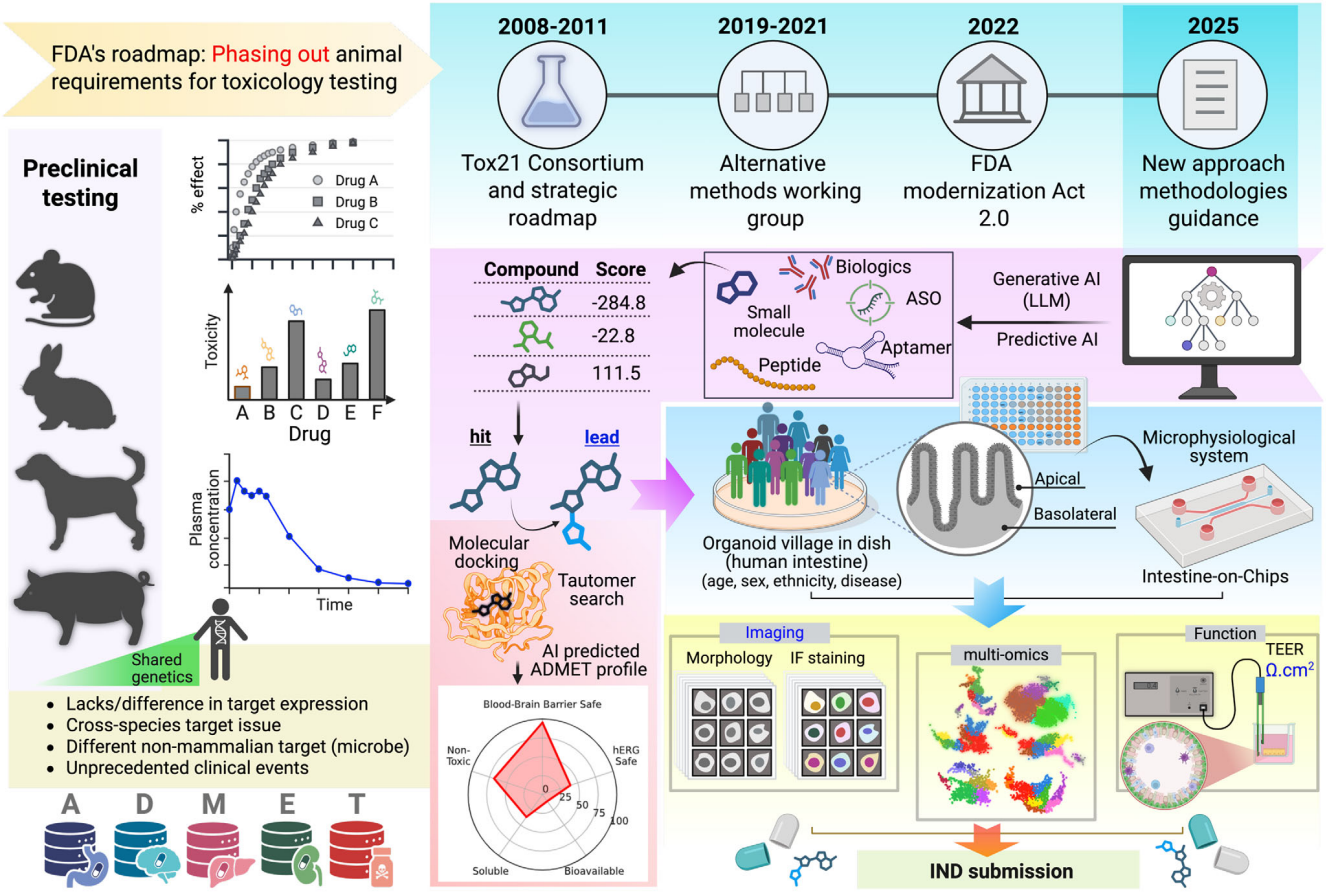
Moving from animal testing to human‐relevant models: how New Approach Methodologies speed up GI toxicity testing. On the left, it shows the problems with animal models. Animals often respond differently from humans because of differences in genes, gut microbes, and drug targets. As a result, animal studies may miss toxic effects or yield misleading safety signals, leading to unexpected problems in clinical trials. The middle and top timelines highlight key FDA milestones that drove this change. It started with Tox21 consortia, followed by alternative testing programs, the FDA Modernization Act 2.0 (2022), which supports the use of finally proposed non‐animal data for regulatory submissions. Finally, in 2025, guidance on New Approach Methodologies (NAMs) was issued to encourage the use of human‐based and computational models. On the right and bottom, the figure proposed a model of how NAMs work in practice. LLMs can be employed for drug discovery for intestinal diseases, which will allow large‐scale screening for drugs to hit specific targets. The top hit can be assessed for its ADMET profile in silico.be screened in silico. Further “Organoid villages,” made from donors of different ages, sexes, ethnicities, and disease backgrounds, will capture human variability for testing drugs in a dish. That can be further integrated into microphysiological systems to simulate real tissue environments. These systems allow detailed testing of how drugs are absorbed, metabolized, and cause toxicity on both sides of the intestinal barrier. In brief, computer‐based tools help screen compounds early by predicting safety and drug‐like properties. In vitro assays in HIOs with those compounds measure intestinal health using molecular profiling, imaging, and functional readouts such as barrier strength. Moreover, these approaches can speed up safer drug development, helping move promising compounds more efficiently toward IND approval. Figure created with BioRender.

Over the past two decades, the FDA has steadily promoted human‐based models to play a larger role in drug safety testing. This started with the Tox21 Initiative, following the Advancing Alternative Methods effort that was aimed at establishing the standards for novel models such as iPSCs and primary cell‐based organoids for drug testing to examine their regulatory implications [112, 113]. A bigger leap forward came in 2022 with the FDA Modernization Act 2.0 promoting the replacement of animal studies with in vitro cell‐based assays and computational approaches to assess the safety and efficacy of biological products [14]. Later updates, such as the FDA Modernization Act 3.0, aim to accelerate the drug discovery process by utilizing animal‐free tests and New Approach Methodologies (NAMs) in preclinical studies [17, 114]. NAMs employ multiple tools such as human‐relevant models (such as organoids), in silico approaches, AI‐based computational methods, and advanced innovative MPS platforms [115] (Figure 3). 3D organoids replicate the human tissue microenvironment and can be utilized with microfluidic devices, such as biofabricated chips, that enable the coculture of epithelial organoids with other disease‐driving cells, including immune cells, fibroblasts, endothelial cells, and microbes [84, 116, 117, 118]. Advanced MPS devices can control the gradient of oxygen and stem and differentiation factors, fluid flow, and mechanical shear stress to recapitulate the human microenvironment [119]. Organoids, including those of the brain, heart, liver, pancreas, kidney, and gut, can be utilized in this system to assess the toxicity and efficacy of drugs on multiple organs simultaneously [120, 121]. In silico approaches and computational tools include the use of mathematical pharmacokinetic models for drug ADME, AI/ML to predict toxicities and off‐target effects, and bioinformatics and quantitative system pharmacology to model the interaction of the drug with human tissues [122, 123, 124, 125]. Intestinal toxicity studies with HIOs, following the FDA's NAMs, open the path for more predictive and accurate preclinical assessment of human drug safety, further reducing the reliance on animal testing.

### 5.1 What Accounts for the Urgency in Regulatory Shifting?

Several reasons underlie the shift in FDA regulatory focus. (1) Animal models do not recapitulate human physiology, and the effects of drugs shown in animal models are significantly different from those in humans. (2) Animal models cannot accurately predict drug outcomes for many chronic diseases, including cancers and neurological diseases. (3) Ethical issues surrounding the use of animals in testing and other studies can be avoided with human‐based approaches. (4) Fundamental differences in drug target expression exist across species, reducing the usefulness of animal models, and (5) non‐mammalian targets such as microbes are also entirely different between humans and animals [1, 15, 126, 127] (Figure 3).

For example, monoclonal antibody development can cost as much as $750 million [128]. Efficacy and toxicity testing of monoclonal antibodies requires a large number of animals and is primarily performed in non‐human primates [129, 130, 131]. However, data from non‐human primates do not align well with human clinical trial data due to significant interspecies differences in immune responses, ultimately contributing to a high failure rate in monoclonal antibody development, which slows the generation of new therapeutics and creates a high cost burden [127]. Additionally, the development process of monoclonal antibodies can take up to 9–20 years [131, 132], and <10% drugs pass the early‐stage development and achieve regulatory approval [132, 133]. The failure rate is particularly high in cases where the etiology of a disease is poorly understood or when a new preclinical mechanistic action of an undrugged target is required [31, 134]. The aforementioned reasons and ethical concerns regarding the use of non‐human primates in specific testing have led the FDA to support the development of new policies and regulations for monoclonal antibody development [135, 136]. The FDA is collaborating with NIH and DVA to implement plans for the next three years, aiming to collect and analyze existing data on drug toxicity worldwide and incorporate it into Investigational New Drug (IND) applications [136]. The FDA will create a free‐access repository to include the known toxicity data and encourage developers to incorporate NAM data into their applications. The FDA aims to reduce the animal test period for drug products from 6 months to 1 month in evaluating the toxicology of monoclonal antibodies [137]. The long‐term goal for the FDA is to make the use of NAMs in all areas of drug safety and efficacy [16, 137]. The use of animal models will remain an alternative in situations where the NAMs cannot provide satisfactory justifications or answers to specific questions or aspects.

### 5.2 So where do Human Intestinal Organoids Fit in?

HIOs are increasingly recognized as powerful tools for early safety assessment. Patient biopsies derived HIOs/PDOs retain patient‐specific genetic and epigenetic states, which are beneficial for studying drug metabolism in physiological conditions [94, 111, 138]. Using HIOs, it is now possible to accurately model interindividual differences in drug metabolism, transporter activity, and immune responsiveness, which are the major determinants of variable toxicity and therapeutic responses observed in human populations [91]. By integrating them with microfluidic flow or organ‐on‐chip designs, HIOs can be exposed to dynamic luminal side to study barrier transport and first‐pass metabolism, further allowing quantitative investigation of toxicokinetics and pharmacokinetics from the assay [84, 117]. Future New Drug Applications (NDA) or biologics submissions will be able to leverage organoid data to explain human‐specific findings (e.g., individuals with IBD or other intestinal diseases), support labeling for gastrointestinal risks, or demonstrate that a compound works safely in a human‐derived context [139].

### 5.3 How do Deep Learning Models and Omics Data Power the Intestinal Drug Discovery Engine?

PDOs generated from healthy or diseased mucosa (e.g., IBD, colorectal cancer), produce rich phenotypic and molecular datasets that exceed the analytical capacity of traditional methods [140, 141]. AI‐driven image analysis, such as D‐CryptO and Deliod, now enables high‐content phenotypic screening of organoids under inflammatory or drug‐treated conditions, extracting quantitative metrics, including crypt morphology, lumen size, and epithelial integrity, in parallel [142, 143]. One recent study demonstrates a scalable imaging‐based readout strategy for nephrotoxicity screening in kidney 3D organoid systems [144]. They combined automated volumetric microscopy with ML‐based image analysis to enable high‐throughput assessment of drug‐induced injury. This pipeline captured complex organoid phenotypes, including structural changes, DNA damage, and injury‐associated morphological features, allowing nephrotoxicity to be evaluated at scale. Similar AI/ML‐based approaches could be adapted for HIOs to perform quantitative analysis of morphology, epithelial dysfunction, barrier breach, and tissue regeneration in response to pathogenic stress or pharmacological treatments. Advanced deep learning frameworks can detect subtle alterations in the morphological signatures of epithelial stress, cytokine‐induced injury, or barrier repair, providing early mechanistic readouts of mucosal protection or toxicity [142, 143, 145, 146, 147]. Multi‐omics (transcriptomic, proteomic, and metabolomics) results generated from HIOs could potentially elucidate the drug response network [148, 149, 150, 151] and help to identify the patient‐specific markers of the drug (e.g., JAK inhibitors) response and resistance [152, 153]. Suggesting, one pipeline that integrates multi‐omics data and morphological phenotypes may enable mapping of drug‐induced responses in intestinal organoids. In the broader preclinical level, these AI‐based analyses are being coupled with high‐throughput organoid screening to accelerate lead optimization for intestinal inflammation and neoplasia [154, 155, 156]. Automated microscopy platforms combined with AI‐based feature extraction can process tens of thousands of organoids per assay, enabling compound prioritization based on phenotype clustering and toxicity prediction [90, 157, 158]. Also, integration of organoid‐derived omics data into in silico ADMET models [125] further refines Point‐of‐Departure estimates and human dose projections.

### 5.4 Can HIOs Qualify for Registration under Clinical Trial Frameworks?

**FIGURE 4:**
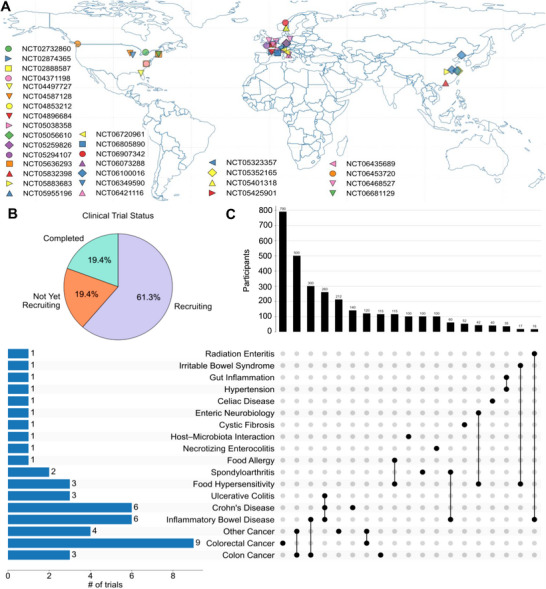
Human intestinal organoids (HIOs) in clinical trials. (A) The global distribution of more than 30 clinical trials listed on ClinicalTrials.gov that use intestinal organoids, along with a world map illustrating trial locations. (B) The pie chart summarizes clinical trial status. (C) The bar and dot plots depict trial distribution by disease type and participant enrollment. Data visualization was performed using Python.

Over 30 registered studies can be found in *clinicaltrials.gov* (Table 3), spanning as many as 10 different intestinal diseases and employing intestinal organoids derived from patients with IBD, CRC, or radiation enteritis to study epithelial regeneration, cytokine signaling, and mucosal repair, among other topics. Among selected studies, 6 are registered in North America, 6 in China, and 18 in Europe. Most of the studies (61.3%) are currently recruiting patients, and approximately 20% have been completed. While other forms of intestinal diseases exist, >1000 patients will be recruited for colorectal cancer (Figure 4). The growing adoption of NAMs and their demonstrated effectiveness for toxicity investigations point to a future in which they will replace traditional animal models using advanced HIOs/PDOs systems. A high‐throughput pharmacological screening on TTC7A knockout cells identified Leflunomide as an anti‐apoptotic compound and improved intestinal survival in PDOs [159]. In the case of rare diseases, organoid‐based drug screening becomes impeccable. In cystic fibrosis, PDOs have been used to test how specific CFTR mutations respond to CFTR modulators before treatment [160, 161]. This approach is known as ‘*theratyping*’ [161]. It is beneficial for rare CFTR mutations where running a traditional clinical trial is not feasible. Utilizing this approach, the FDA approved a CFTR modulator, ivacaftor, for patients with CFTR mutations that are responsive to potentiation [162]. Organoid testing helped identify that elexacaftor/tezacaftor/ivacaftor could work for rare CFTR variants such as N1303K, T465N, L1065P, and R1066H, allowing patients with these mutations to receive treatment [163, 164, 165]. Other than GI disease, NAMs' based disease modeling platform mediates preclinical to clinical transition effectively. In the case of Amyotrophic lateral sclerosis (ALS), iPSC‐neurons used for drug screening led to the discovery of ropinirole [166]. This drug inhibits motor neuron degeneration by suppressing cholesterol biosynthesis. A recent double‐blind, phase 1/2a trial of ropinirole was conducted in 20 participants with sporadic ALS [167], providing clear evidence that human iPSC‐based NAMs can inform preclinical decision‐making.

**TABLE 3:** Clinical trials utilizing human intestinal organoid models for drug testing and studying mechanisms.

Disease	Trial objective	Status	Source	Endpoints	Type	Size (n)	Location	NCT Number
Inflammatory Bowel Disease (Crohn's & UC)	Develop and characterize patient‐derived intestinal organoids to model epithelial regeneration and disease‐specific responses	Recruiting	Intestinal biopsies	Yield of intestinal crypts, differentiation efficiency, expression of MUC2, LGR5, and KI67	Interventional	90	France	NCT05294107
Inflammatory Bowel Disease (Crohn's & UC)	Assess epithelial repair mechanisms and regeneration deficits in IBD using patient‐derived intestinal organoids exposed to inflammatory cytokines and metabolites	Recruiting	Colonoscopy and surgical biopsies (inflamed vs. non‐inflamed mucosa)	Organoid proliferation, survival, and differentiation (Ki67, LGR5, MUC2); evaluation of cell death pathways and repair signatures	Interventional	60	France	NCT06805890
Inflammatory Bowel Disease (Crohn's & UC)	Characterize intestinal stem cells and Wnt/APC/β‐catenin signaling using patient‐derived intestinal organoids from IBD, FAP, and healthy controls	Completed	Endoscopic biopsies	Organoid count, mean size, and morphology; Wnt pathway gene/protein expression	Interventional	110	France	NCT02874365
Visceral Hypersensitivity / Enteric Neurobiology	Examine innervation and sensory receptor activity in gut‐derived enteroendocrine cells using human intestinal organoids	Completed	Endoscopic and colonoscopic biopsies	Electrophysiological excitability and sensory receptor expression in enteroendocrine cells; correlation with RNA‐seq	Observational	42	USA	NCT02888587
Spondylo‐arthritis / Inflammatory Bowel Disease	Develop and validate patient‐derived intestinal and synovial organoid models to study the gut–joint axis and identify inflammatory biomarkers	Recruiting	Synovial and intestinal biopsies	Organoid viability (ATP assay), cytokine secretion (ELISA), and fibroblast invasion capacity	Observational	60	Italy	NCT06421116
Hypertension / Gut Inflammation	Compare gut epithelial gene expression and organoid growth profiles between hypertensive and normotensive subjects using patient‐derived colon organoids	Completed	Colonic biopsies	Differentially expressed epithelial genes (RNA‐seq) and organoid growth rates between groups	Observational	35	USA	NCT04497727
Radiation Enteritis / Inflammatory Bowel Disease	Validate organoid‐based preclinical platform to assess intestinal regeneration after irradiation and test therapies for IBD and radiation injury	Completed	Ileocolon‐oscopic biopsies (healthy & IBD mucosa)	Organoid viability, growth, composition, apoptosis, cytokine levels before/after irradiation and treatment	Interventional	16	France	NCT05425901
Inflammatory Bowel Disease / Colon Cancer	Build a biobank of blood and intestinal biopsies with clinical data to study gut disease and test therapies on organoids	Recruiting	Blood and intestinal biopsy samples	Establish biological collection with clinical data; evaluate drug effects on intestinal organoids	Observational	300	France	NCT04896684
Irritable Bowel Syndrome (IBS) / Food Sensitivity	Assess effect of food antigens on intestinal mucosal integrity	Completed	Duodenal biopsy	Correlation of CLE findings with histopathology and organoid responses	Interventional	17	Germany	NCT05056610
Host–Microbiota Interaction / Immunology	Investigate host–microbe and metabolite interactions using human intestinal organoids	Recruiting	Intestinal biopsy	Transcriptomic and epigenomic profiling before and after microbial exposure	Observational	100	Switzer‐land	NCT05323357
Food Allergy / Food Hypersensitivity	Diagnosis of food allergy by correlating mucosal IgE and inflammation with organoid	Recruiting	Duodenal biopsies	Correlation of mucosal IgE with gene and protein expression in allergen‐stimulated organoids	Interventional	115	Germany	NCT05259826
Rectal Adeno‐carcinoma	Evaluate feasibility of generating organoids from pre‐treatment rectal cancer biopsies	Completed	Tumor biopsy	Success rate of organoid formation from patient samples	Interventional	20	USA	NCT04371198
Advanced Rectal Cancer	Compare efficacy of organoid‐guided drug‐sensitive neoadjuvant therapy	Not Yet Recruiting	Tumor biopsies from rectal cancer	Pathologic complete response, recurrence rate, and R0 resection rate	Interventional (Randomized Controlled)	192	China	NCT05352165
Advanced / Recurrent Colorectal Cancer	Use PDOs and PDXs to identify actionable molecular targets and predict drug response for precision therapy	Not Yet Recruiting	Tumor biopsies	Identification of effective drugs and correlation of genomic and phenotypic responses	Observational	100	China	NCT05883683
Colorectal Cancer	Assess concordance between clinical efficacy and organoid‐based drug susceptibility testing in CRC patients	Recruiting	Tumor biopsies, surgical or effusion samples	Correlation between in vitro drug sensitivity and patient clinical response	Observational	105	China	NCT06100016
Stage IV Colorectal Cancer	Evaluate whether organoid‐guided chemotherapy improves outcomes compared to standard regimens	Recruiting	Tumor biopsies	Progression‐free and overall survival	Interventional	186	China	NCT05832398
Cystic Fibrosis (CF)	Validate organoid‐based CFTR modulator response predictions in patients with rare CFTR mutations	Recruiting	Intestinal organoids from CF patients	Lung function (ppFEV_1_), sweat chloride, body weight, CFQ‐R respiratory domain	Interventional (Phase II, Randomized Crossover)	52	Multi‐center EU trial	NCT06468527
Spondylo‐arthritis	Role of Ruminococcus gnavus and intestinal dysbiosis in spondyloarthritis	Recruiting	Intestinal biopsies, fecal samples	Comparative microbiota profiling, RNA‐seq of biopsy, and host–microbe interaction assays	Interventional	100	France	NCT04853212
Crohn's Disease (Intestinal Fibrosis)	Investigate microbiota‐driven mechanisms underlying intestinal fibrosis in Crohn's disease	Not Yet Recruiting	Surgical specimens, isolated cell populations	Multi‐omics profiling (RNA‐seq, metatranscriptomics, lipidomics) to link microbial factors to profibrotic pathways	Observational (Mechanistic Research)	20	Italy	NCT06073288
Necrotizing Enterocolitis (NEC)	Characterize human intestinal development and disease mechanisms in neonates	Not Yet Recruiting	Neonatal intestinal biopsies	Cellular composition, immune markers, and microbiome characterization	Observational	100	USA	NCT06681129
Crohn's Disease (Intestinal Fibrosis)	Identify and characterize bacterial factors driving gut fibrosis	Not Yet Recruiting	Surgical specimens, stool samples	Multi‐omics profiling and validation in fibrosis organoid and animal models	Observational	20	Italy	NCT06720961
Colon Cancer	Evaluate CD47–SIRPα inhibitors on the immune microenvironment	Recruiting	Blood and tumor biopsies	Establishment of organoid and T‐cell biobanks; assessment of immunotherapy‐induced tumor viability	Observational	115	France	NCT05955196
Crohn's Disease	Determine if gut microbiome and organoid‐derived epithelial profiles can predict treatment response in IBD	Recruiting	Fecal, intestinal washings, and biopsy‐derived organoids	Microbial and host transcriptomic correlations with therapy outcomes over 12 months	Observational	100	Canada	NCT06453720
Colorectal Cancer	Effects of high‐fiber/low‐fat preoperative diet on gut microbiome, tumor microenvironment	Recruiting	Stool, blood, and tumor resection	Changes in collagenase activity, microbiome composition, and metabolic profiles	Interventional (Phase I/II)	80	USA	NCT06349590
Colorectal Cancer	Develop personalized ex vivo organoid assays to predict response to chemo‐ and CAR T cell–based therapies	Recruiting	Tumor tissue	Organoid development success rate and drug sensitivity correlation with immunotherapy efficacy	Observational	40	Norway	NCT05401318
Colon & Metastatic Peritoneal Cancer	Characterize and modulate immune responses in primary and metastatic CRC	Not Yet Recruiting	Tumor and mucosal samples	Evaluation of immune–tumor co‐culture response, transcriptomic/proteomic/TCR repertoire profiling	Observational	500	France	NCT06435689
Colorectal Cancer	Develop patient‐derived tumoroid models to study immune‐mediated chemoresistance and identify predictive biomarkers	Recruiting	Tumor biopsies	Organoid viability over 60 days and correlation with immune and resistance signatures	Observational	60	France	NCT05038358
Colorectal, Breast & Ovarian Cancers	Establish personalized patient‐derived xenograft (pPDX) and organoid models to predict individual drug responses	Recruiting	Tumor biopsies	Correlation between pPDX drug sensitivity and patient response; engraftment rate and genomic concordance	Observational	120	Canada	NCT02732860
Metastatic Colorectal Cancer	Evaluate a functional precision medicine platform using patient‐derived tumoroids	Recruiting	Core needle tumor biopsies	Feasibility of generating valid tumoroid response reports; correlation with clinical outcomes (ORR, PFS, OS)	Interventional (Phase II)	148	Norway	NCT06907342
Celiac Disease	Assess safety and efficacy of Ritlecitinib during gluten challenge in patients with celiac disease in remission	Recruiting	Intestinal biopsies, blood, stool	Change in villus height: crypt depth ratio, gluten‐specific T‐cell and cytokine profiles, intestinal organoid modeling	Interventional (Phase II, Double‐Blind, Placebo‐Controlled)	40	USA	NCT05636293
Metastatic Colorectal Cancer	Evaluate early‐line EGFR inhibitor therapy (cetuximab/panitumumab) and explore organoid‐predicted response and resistance mechanisms	Recruiting	Tumor biopsies	Disease control rate, PFS, OS, retreatment rate, organoid‐predicted response to EGFR inhibition	Interventional (Phase II)	71	USA	NCT04587128

*Source*: October 2025, *ClinicalTrials.gov* database.

### 5.5 Case Report: HIOs as a Therapeutic

PDOs cultured in a dish become a game changer, particularly with their potential in regenerative medicine [139, 168]. Previous studies in the Dextran Sulphate Sodium salt (DSS)‐induced ulcerative colitis mouse model found that autologous transplantation of intestinal organoid not only restored the epithelial integrity, but also precisely homed in on the injured epithelium while improving clinical scores [169]. Building on these results, PDOs were transplanted into the colonic mucosa of a patient with refractory ulcerative colitis at the Tokyo Medical and Dental University, Japan [170]. They collected adult stem cells from intestinal biopsies of healthy individuals, cultured them into 3D spheroids (0.1–0.2 mm), and delivered them endoscopically to the lesion site [171]. The follow‐up endoscopy showed mucosal repair and an improvement in lesion appearance, establishing clinical proof of concept for organoid‐based regenerative therapy [27].

## 6 Challenges and Opportunities

HIOs come with a transformative potential to serve as NAMs to act as a model of a highly human‐specific predictive and standardized tool for preclinical drug discovery [172]. Completely replacing in vivo testing will take time, as challenges such as context‐specific standardization, technical, and translational limitations need to be addressed before widespread adoption of the model [173]. One limitation of HIOs, differentiated from intestinal adult stem cells, is that they are primarily composed of epithelial cells and lack immune, vascular, and microbial components that contribute to shaping intestinal physiology and disease [174]. Compositional simplicity restricts their ability to mimic inflammatory, fibrotic, or immune‐mediated gut pathologies such as IBD and colorectal cancer [175]. However, their physiological or disease relevance can be further enhanced by placing them in co‐culture platforms that integrate immune cells, fibroblasts, and patient‐derived microbiota, and iPSC‐derived HIOs have stromal cells, including vasculature and neurons [65, 176]. Microfluidic “organ‐on‐a‐chip” systems can further enable dynamic modeling of fluid flow, nutrient gradients, and host–microbe crosstalk, offering a more physiologically faithful in vitro environment [177]. Absence of functional vasculature represents another bottleneck, as vascularization is essential for long‐term viability of the 3D organoids and their maturation. Vascularized organoid‐on‐chip systems of kidney [178], liver [179], and pancreas [180], among others, have been demonstrated to overcome the diffusion limitations inherent to static organoid cultures by culturing under flow conditions. These chip platforms enhance the viability and differentiation of these organoids, thereby potentiating translational relevance in studies modeling drug toxicity and tissue responses. Suggesting the integration of perfusable microvascular networks into gut organoids with peristaltic movement, fluids, and oxygen control, and exposure to microbes and immune cells will meaningfully mimic the human gut when cultured in a chip.

Reproducibility may be a bigger obstacle in drug discovery, as drug responses are heterogeneous among PDOs due to true biological diversity [181]. Variations in growth media composition, Matrigel batches, and culture conditions can introduce additional confounding effects [182, 183]. The introduction of serum‐free, fully defined cell culture media aims to reduce variability and improve reproducibility [184]. Also, the extracellular matrix problem was further addressed by emerging animal‐free scaffolds, including recombinant polymers produced by bacteria, plant‐derived biocompatible scaffolds, nanomaterials, engineered synthetic polymers, and decellularized human tissues [185, 186, 187]. Recent work by Wijnakker et al. has demonstrated that synthetic polyisocyanide hydrogels having the integrin‐activating domain of Invasin can reliably support epithelial organoid growth while minimizing batch effects [188]. Not only does this approach improve reproducibility, but it also standardizes animal‐free scaffolds in 3D organotypic models, which emphasize the value of engineered matrices for reproducible, ethically responsible culture systems. Further improvement can be augmented by using early‐passage ISC‐derived organoids to preserve disease‐relevant phenotypes in vitro [29]. Peptide‐based hydrogel (PeptiMatrix 7.5) also shown promising results for hepatocytes, with improved metabolic function, albumin secretion, and CYP3A4 activity under perfused conditions [189]. In‐parallel, recent efforts have been committed to create a biobank in multiple centers across the globe that preserve and store biopsies derived from stem cell organoids, and iPSCs that encompass sex, age, race, ethnic backgrounds, and disease status are now available; when supported by standardized culture protocols and open‐access data repositories, these advances make possible a novel “clinical trial in a dish” approach creating “organoid village” platforms that are connected with MPS to enable population‐scale screening, thereby improving predictive accuracy in precision pharmacology [68, 190, 191, 192].

While HIOs replicate intestinal absorption and barrier functions, they cannot independently model systemic pharmacokinetics, metabolism, and excretion, which are governed by other organs [193]. Coupling intestinal organoids with liver, kidney, or brain organoids through MPS will improve modeling of the gut–liver, gut–kidney, and gut–brain axes that are critical for understanding drug distribution, first‐pass metabolism, and off‐target toxicity [81, 194, 195]. The FDA's roadmap toward adopting integrated NAMs highlights these cross‐organ systems as the future of human‐relevant pharmacological modeling [16].

Other challenges existed with closed organoid lumens for drug exposure studies, and upscaling organoid assays for high‐throughput applications requires engineering innovation [92, 196]. Whereas a much simpler 2D platform in a transwell or on a chip can open both the apical and basolateral sides, making them suitable for functional drug action [197, 198]. Translation advancement can be performed by integrating deep learning model‐driven imaging analysis, multi‐omics profiling, and machine learning‐based predictive modeling. Such integration can transform these platforms into quantitative tools for toxicokinetic assessment, dose prediction, and biomarker discovery. AI‐based outputs can be further fed into physiologically based pharmacokinetic models to apply in vitro data to human‐equivalent doses, which will be effective in understanding the relationship between in vitro data and human exposure [199]. Additionally, HIOs are utilized for studying the pharmacodynamics of drugs, including drug‐drug interactions, drug‐receptor interactions, dose‐response relationships, and therapeutic indices [91].

HIOs (iPSC‐derived or PDOs) are becoming essential for the study of monogenic intestinal diseases such as cystic fibrosis, as they retain the patient's original genetic background and pathogenic signature [200]. This utility is seen in the case of CFTR mutation organoids that fail to transport Cl‐ ion, leading to impaired fluid homeostasis [201, 202, 203]. By using CRISPR‐based gene editing, normal channel function and swelling responses can be restored [203, 204, 205, 206]. This breakthrough not only validated the organoid system as a functional disease model but also demonstrated its potential for personalized gene‐correction therapy. Combining powerful genome editing with more standardized and efficient protocols will help make intestinal organoids routine preclinical testbeds for evaluating gene therapy efficacy, as well as autologous grafts for regenerative treatment of intractable hereditary intestinal disorders in the future.

FDA's remarkable regulatory shift over the last three years has led to the acceptance of NAMs‐based drug discovery at the nonclinical level [1, 16, 135]. It will take time for a complete transition to take effect with the buy‐in of stakeholders, including the pharmaceutical industry and academic researchers, as they move away from the use of animals in drug safety studies. To fully realize the promise of organic‐based NAMs, collaborative efforts among academia, industry, and regulatory agencies are needed to establish shared standards for reproducibility, scalability, and data integration. Amongst other requirements, it will be necessary to establish context‐specific shared standards for toxicity assays and quality control pipelines that validate intestinal organoids (as well as other NAMs) [207] as reliable surrogates for human tissues in safety and efficacy evaluations.

## Conflicts of Interest

The authors declare no conflicts of interest.

## Data Availability

The authors have nothing to report.
